# The Design and *In Vivo* Evaluation of Engineered I-OnuI-Based Enzymes for HEG Gene Drive

**DOI:** 10.1371/journal.pone.0074254

**Published:** 2013-09-10

**Authors:** Yuk-Sang Chan, Ryo Takeuchi, Jordan Jarjour, David S. Huen, Barry L. Stoddard, Steven Russell

**Affiliations:** 1 Department of Genetics, University of Cambridge, Cambridge, Cambridgeshire, United Kingdom; 2 Division of Basic Sciences, Fred Hutchinson Cancer Research Center, Seattle, Washington, United States of America; 3 Northwest Genome Engineering Consortium, Seattle, Washington, United States of America; 4 Pregenen Inc., Seattle, Washington, United States of America; 5 Cambridge Systems Biology Centre, Cambridge, Cambridgeshire, United Kingdom; National Cancer Institute, United States of America

## Abstract

The homing endonuclease gene (HEG) drive system, a promising genetic approach for controlling arthropod populations, utilises engineered nucleases to spread deleterious mutations that inactivate individual genes throughout a target population. Previous work with a naturally occurring LAGLIDADG homing endonuclease (I-SceI) demonstrated its feasibility in both *Drosophila* and *Anopheles*. Here we report on the next stage of this strategy: the redesign of HEGs with customized specificity in order to drive HEG-induced ‘homing’ *in vivo* via break-induced homologous recombination. Variants targeting a sequence within the *Anopheles AGAP004734* gene were created from the recently characterized I-OnuI endonuclease, and tested for cleavage activity and frequency of homing using a model *Drosophila* HEG drive system. We observed cleavage and homing at an integrated reporter for all endonuclease variants tested, demonstrating for the first time that engineered HEGs can cleave their target site in insect germline cells, promoting targeted mutagenesis and homing. However, in comparison to our previously reported work with I-SceI, the engineered I-OnuI variants mediated homing with a reduced frequency, suggesting that site-specific cleavage activity is insufficient by itself to ensure efficient homing. Taken together, our experiments take a further step towards the development of a viable HEG-based population control strategy for insects.

## Introduction

The ability to design gene-specific endonucleases against custom DNA sequences (recently reviewed in [1]) is an essential component for the targeted gene modification underpinning some proposed insect eradication strategies. While a number of such gene targeting nuclease scaffolds exist, including zinc-fingers (ZFNs) [2], TALENs [3], and the CRISPR/Cas9 system [4], [5], LAGLIDADG homing endonuclease genes (‘HEGs’) encode gene targeting proteins that offer the advantage of a naturally occurring, compact monomeric architecture [6]. Directed searches of microbial genomes have uncovered large families of putative homing endonuclease genes, a subset of whose recognition sites can be predicted and validated based on the sequence surrounding a homing site [7], [8]. We have successfully demonstrated that an endonuclease variant redesigned from a representative of the novel monomeric LAGLIDADG HEGs, I-OnuI, induced targeted mutagenesis at the human endogenous gene locus [7].

The HEG drive strategy proposes exploiting the biochemical activity and homing behaviour of HEGs to drive targeted gene disruption through an arthropod pest population [9]. This approach has shown promise in initial evaluations with both *Drosophila melanogaster* and the malaria vector, *Anopheles gambiae* [10]–[12]. Considerable development is currently directed towards deploying the strategy for the control of the latter species. In models of HEG drive systems, it is strategically advantageous to target female-specific genes required for fertility [13], [14] and a panel of putative *Anopheles gambiae* female germline-specific genes was identified using bioinformatics approaches [15]. Screening this panel against a library of known target sites for naturally-occurring LAGLIDADG homing endonucleases yielded a number of hits that were subsequently ranked by predicted ease of creating redesigned HEGs [16]. A DNA sequence in *AGAP004734*, which only differs from the canonical I-OnuI target site at seven positions, was chosen for priority development because of the extensive experience we have accumulated in engineering the I-OnuI scaffold. Here we report the characterisation of four I-OnuI-derived endonuclease variants that specifically target a sequence in the *AGAP004734* gene using our previously described *Drosophila in vivo* model system [10], [12].

## Materials and Methods

### Assembly of Active I-OnuI Variants using Yeast Surface Display

I-OnuI variants cleaving the *Anopheles AGAP004734* target were isolated using multiple rounds of site-directed saturation mutagenesis (to alter specificity of the wild type I-OnuI) and selection of active endonuclease variants displayed on the surface of yeast by fluorescence-activated cell sorting (FACS) [17]. Briefly, *Saccharomyces cerevisiae* (EBY100 strain) were transformed using the lithium acetate method with a linearized, I-OnuI encoding plasmid and short DNA fragments containing partial I-OnuI gene sequences with NNS codons at positions to be randomized. These wobble bases were introduced by PCR from synthesized oligonucleotide templates. Residues on the protein-DNA interface that were likely to participate in recognition of the wild type I-OnuI target site DNA sequence (by making contacts with DNA bases or backbones or by interacting with other side chains) were mutated. Cleavage activity of variant endonucleases was detected by reduced fluorescence (Alexa-647) signal due to release of cleaved products, as described previously [17]. In the first round of screening, yeast cells expressing variant endonucleases that cleaved a DNA substrate containing a local stretch of sequence from the *AGAP004734* target integrated into the wild type I-OnuI site (see Figure S1 in File S1 for details) were collected by FACS after an *in vitro* cleavage reaction that ran for 20 minutes in 150 mM KCl, 10 mM NaCl, 10 mM HEPES, 5 mM potassium glutamate, 0.5% BSA, 5 mM MgCl_2_. Plasmids were recovered from the active populations (Zymoprep II, ZymoResearch) and subjected to sequencing. Secondary libraries were designed based on sequence information from clones selected through the first round of screening and used to identify active variant genes against each half of the *AGAP004734* target linked to the other half of the wild type I-OnuI target. Yeast cells displaying active variants were sorted again as described above and each half domain containing amino-acid substitutions resulting from site-directed saturation mutagenesis were assembled to construct a library for selection of variants that cleaved the full *AGAP004734* target site. The I-OnuI variants cleaving the *AGAP004734* target were sorted and sequenced.

### Optimization of I-OnuI Variant Activities using a Bacterial Two-plasmid Cleavage Assay

The activity of I-OnuI variants generated using the yeast surface display selections was further optimized using a two-plasmid selection system in bacterial cells [18]. To obtain variants of I-OnuI that efficiently cleave the *AGAP004734* gene target, the N-terminal and C-terminal half protein domains of eight unique clones from the final yeast sort were shuffled by overlapping PCR and inserted between *Nco*I and *Not*I sites of the pEndo expression plasmid. NovaXGF (Novagen) competent cells harbouring the pCcdB reporter plasmid (containing 4 copies of the *AGAP004734* gene target) were transformed with the pEndo plasmid encoding I-OnuI variants. The transformants were grown in 2×YT medium (16 g/L tryptone, 10 g/L yeast extract, and 5 g/L NaCl) at 37°C for 30 min and then diluted 10-fold with 2×YT medium supplemented with 100 µg/mL carbenicillin and 0.02% *L*-arabinose (in order to preinduce expression of I-OnuI variants). After the culture was grown at 30°C for 15 hours, the cells were harvested, resuspended in sterile water and spread on both nonselective (1×M9 salt, 1% glycerol, 0.8% tryptone, 1 mM MgSO4, 1 mM CaCl2, 2 µg/mL thiamine, and 100 µg/mL carbenicillin) and selective plates (the **nonselective** plates supplemented with 0.02% *L*-arabinose and 0.4 mM IPTG to induces expression of the toxic CcdB protein). After incubation at 30°C for 30–40 hours, the pEndo plasmid was recovered from the surviving colonies on the selective plates.

The ORFs encoding active I-OnuI variants were amplified via error-prone PCR using the Gene Morph II Random Mutagenesis Kit (Agilent Technologies). After digestion with *Nco*I, *Not*I and *Dpn*I, the resulting fragments were recloned into the pEndo vector. The plasmid was subjected to 2 rounds of selection under conditions where variant endonucleases were expressed at 30°C for 4 hours before plating. The selected ORFs were again cloned into the pEndo vector and used for selection at higher stringency. Transformed cells carrying both the pEndo plasmid and the pCcdB reporter were grown in 2×YT medium containing 0.02% *L*-arabinose at 37°C for an hour and then screened on selective plates at 37°C for 16–20 hours. The pEndo plasmid was extracted from colonies on the selective plates and ORFs of the variant genes carried on the plasmid were sequenced.

### *in vivo* Homing Assay

The *Drosophila in vivo* homing assay has been previously described in detail [10]. The assay uses the ΠC31 integrase system to place donor constructs expressing a HEG and target constructs containing the HEG recognition site at homologous locations in the *Drosophila* genome [19]. A graphical representation of the assay is reproduced in Figure S2 in File S1 (originally published in [12]). As the HEG transcription unit is marked by mRFP1 and the target site is within an eGFP ORF that is expressed in the eye, homing of the HEG to the target site can be readily followed by RFP fluorescence. Disruption of the target site by NHEJ is also detectable by loss of the GFP fluorescence in approximately two-thirds of the cases. We previously showed that a design based on the *Rcd-1r* promoter and the *β-Tub56D* 3′-UTR had the best homing performance when comparing a panel of different construct designs [12]. The constructs used in this study are modifications of the HEG-2 constructs we previously described, replacing the I-SceI recognition site with the *AGAP004734* target site. For reasons previously described, we elected to use the directly-measurable GFP loss (the ratio of GFP-negative target-bearing progeny count to the total target-bearing progeny count) and homed fraction (the fraction of GFP-negative targets derived from homing events) as proxies for HEG activity and homing efficiency respectively [12].

Bulk crosses were performed in bottles with 5–15 transheterozygote males and ∼20 females. Each experiment usually consisted of three bulk crosses with experiments performed on two separate occasions and all data from the 5–6 bottles for each HEG was combined.

As the *Rcd-1r* promoter drives specific expression in spermatogonia, all repair events can be expected to be pre-meiotic resulting in clusters of 2–16 spermatids that inherit the same lesion. Progeny genotypes are therefore not independently drawn events with consequent loss of statistical power. We are not aware of any treatment that specifically handles this case and have therefore adopted the expedient of deflating all counts by an effective cluster size and rounding down the resultant values before they were used for statistical purposes. This approach provides an approximate estimate of the actual event counts and since the actual cluster size is not known, a conservative cluster size of 16 was selected (i.e. the event is assumed to have occurred in the spermatogonium immediately after asymmetric division of the germline stem cell, resulting to the largest cluster size) and it should lead to a conservative estimate of the statistical significance of comparisons performed in this study.

Given that HR occurs only in the spermatogonia in *Drosophila melanogaster*, this issue may extend to any experiment investigating HR using progeny counts in this species.

### Characterisation of Repair Products

GFP^−^ RFP^−^ progeny were analysed as previously described [6]. The replacement of the I-SceI target site with one derived from *AGAP004734* did not materially affect the assay: PCR amplification with the eGFP-L2/eGFP-R2 primer pair resulted in a fragment of 613 bp. The assay was extended with two further primers, mRFP-2 (CTC GAA CTC GTG GCC GTT CA) and Rcd-1r-2 (CCG GTG GGT CAT GTT ATG GT), to permit identification of incomplete homologous recombination products. The eGFP-L2/mRFP-2 and Rcd-1r-2/eGFP-R2 primer pairs amplify the left and right junctions of the homing unit respectively (Figure S3 in File S1). Sequence alignments were performed with Clustal-Omega and manually refined [20]. Where the sequence could be aligned in multiple ways, alignments that preserved the ATTC overhang at the cleavage site were chosen.

## Results and Discussion

### Design of I-OnuI to Target the Anopheles Gene Sequence

The candidate *AGAP004734* target site differs from the 22 bp I-OnuI recognition site at 7 positions (Table 1). Using yeast surface display technology, we isolated nine initial I-OnuI variants that were capable of cleaving the *AGAP004734* target. These genes were then subjected to random mutagenesis followed by selection in bacteria to increase cleavage activity (see methods for details). In bacterial cells, two distinct endonuclease genes, termed ‘H4734A’ and ‘H4734B’, displayed activity similar to the previously engineered I-OnuI variant that cleaved the endogenous human *monoamine oxidase B* gene (1). The new variants contain 17 and 15 amino-acid substitutions, respectively, relative to their parental enzyme, most of which were located within peptide loops connecting two β-sheets in the vicinity of each end of the DNA target site (Figures S4 and S5 in File S1). Of the residues that were altered in each of the two enzyme constructs, seven positions harboured the same mutation in both engineered HEGs. Two of the shared substitutions were introduced on the protein-DNA interface; two others on a loop of the C-terminal half domain; the rest relatively distant from the DNA binding surface, but proximal to one another. Note that the parental endonuclease used to generate the two *AGAP004734* gene targeting nucleases already possessed four individual amino-acid substitutions (relative to the true wild type I-OnuI) that were incorporated to improve expression levels prior to the protein engineering process (Figure S4 in File S1).

**Table 1:** Activity of parental and variant endonucleases in bacteria.

	Target sites^1,2^		
Endonuclease		I-OnuI	AGAP004734 gene
I-OnuI		98%	<0.010%
Inactive I-OnuI (E22Q)		<0.010%	n.d.*
H4734A		0.049%	40%
H4734B		55%	56%

1 Values indicate the percentage of survival rate, which is calculated by dividing the number of colonies on the selective plates by that on the nonselective plates.

* not determined.

2 The recognition sites of I-OnuI and the AGAP004734 target site are:

***AGAP004734*** **T**g**TCCAC**ac **ATTC** **AA**a**CTT**aac.

**I-OnuI** T TT C TTA.

In the two-plasmid cleavage assay employed in the present study, transformation with an expression plasmid for I-OnuI rescued bacterial cells that harbour the pCcdB reporter containing the I-OnuI target sites on the selective medium plates where expression of the toxic DNA gyrase inhibitor gene was induced (Table 1). In contrast, the catalytically inactive I-OnuI variant with a single amino acid substitution in its active site (E22Q) failed to support cell growth under the same conditions, indicating that hydrolysis of a target site on the pCcdB reporter plasmid was essential for cell survival on the selective plates. In this assay, H4734B displayed slightly higher activity against the *AGAP004734* target than H4734A, but cleaved the original wild-type I-OnuI target with similar efficiency (Table 1). In contrast, H4734A preferentially cleaved the *AGAP004734* target relative to the I-OnuI target, while retaining levels of activity comparable to H4734B, thus appearing to retain greater site discrimination ability than H4734B. The H4734A and H4734B I-OnuI variants were both further engineered by introducing a previously identified active site mutation (E178D) that was likely to increase overall catalytic efficiency, generating enzymes designated as H4734A* and H4734B* [7].

To evaluate the efficacy of the engineered HEGs in an insect *in vivo* gene drive context, H4734A and H4734B and their derivatives with the E178D substitution were assayed using our previously described *Drosophila* model [10]. Transgenic donor lines containing each of the four HEGs and a recipient line containing the *AGAP004734* target within the GFP ORF were derived by φC31 integration at the *attP2* site on Chromosome *3L*. While the H4734A and H4734B lines were derived without difficulty, the first generation male founders derived from both E178D variants were observed to be sterile for several days after eclosion but eventually became fertile and stocks derived from them could be maintained under laboratory conditions, albeit requiring greater care. Reduced fertility was also occasionally observed in subsequent crosses where E178D variant lines were used to set up trans-heterozygotes with the target sequence lines. We speculate that the initial sterility arose as a result of more promiscuous activity by E178D variants, creating double-strand breaks at genomic loci other than the recipient target. Since these break sites can be repaired by nonhomologous end joining (NHEJ) that frequently induces short sequence deletions and insertions, leading to disruption of sites cleavable by engineered HEGs, we suspect that only such clones eventually dominated the male germline. The transmission of these non-cleavable sites to progeny eventually result in more fertile transgenic E178D lines.

### HEG Activity and Homing Performance *in vivo*

A brief description of the *in vivo* assay is available in Figure S2 in File S1. We observed that all HEGs showed cleavage activity against the AGAP004734 target in the *in vivo Drosophila* assay, but, in contrast to the *in vitro* assay where the H4734A variant was less active than H4734B, both variants showed similar activity *in vivo*. However, both H4734A and H4734B were considerably less active than I-SceI in our *Drosophila* assay, yielding less than half the GFP loss induced by the latter when driven by the same regulatory elements at the same chromosomal location. In line with expectations, the inclusion of the E178D substitution increased activity to a level corresponding to near-complete loss of GFP expression, presumably through increased enzymatic activity, although other mechanisms are formally possible, e,g, a shift from religation to HR/NHEJ-mediated repair.

Using fluorescent markers as a readout, all four of the engineered HEGs showed a lower homed fraction than I-SceI when assayed under the same conditions, having half the homing fraction of I-SceI (Table 2, row 2). The homing fraction was not significantly different among I-OnuI transgenic lines (Fisher’s p-value = 0.48). However, the fraction of RFP^+^ (as a marker of homing) progeny from the H4734A*^−^ or H4734B*^−^ expressing parents was comparable to that from the I-SceI-expressing line (Table 2, row 3), because these two engineered I-OnuI variants appeared to cleave their target site more effectively than I-SceI.

**Table 2:** Results for AGAP004734-target HEGs.

	I-SceI^1^	H4734A	H4734A*	H4734B	H4734B*
	Percentage[CI](Counts)	Percentage[CI](Counts)	Percentage[CI](Counts)	Percentage[CI](Counts)	Percentage[CI](Counts)
GFP−/total	37 [31–44](1273/3422)	16^2^ [11]–[21](509/3251)	87^2,3^ [83–91](3682/4218)	13^2^ [9]–[19](362/2860)	86^2,3^ [79–90](2007/2347)
Homed/GFP-	61 [50–71](782/1273)	33^2^ [19–50](168/509)	29^2^ [24–35](1077/3682)	30^2^ [13–48](107/362)	22^2^ [16–30](451/2007)
Homed/total	23 [17–29](782/3422)	5^2^ [3]–[8](168/3251)	26 [21–31](1077/4218)	4^2^ [2]–[7](107/2860)	19 [14–26](451/2347)

1 Previously reported in [10].

2 Different from equivalent I-SceI value (Fisher’s exact test p-value<0.02).

3 Different from equivalent non-E178D value (Fisher’s exact test p-value<0.02).

95% confidence intervals are listed above.

All calculations were based on the conservative assumption described in methods.

The lesions at the cleavage site of GFP^−^, RFP^−^ progeny (i.e. cases of presumptive NHEJ repair) were characterised by PCR amplification and sequencing. The majority of lesions were typical of HNEJ repair, with micro-deletions at the cleavage site (Figure S6 and Table S1 in File S1). Most micro-deletions appeared to occur on either side of the ATTC overhang sequence with that sequence preserved intact, although this interpretation could be an artefact of the alignment procedure in some cases. In the few cases where micro-deletions were absent, the region around the cleavage site was either duplicated or a few bases were inserted from an unknown template. Oversized PCR fragments were very occasionally obtained (Table S1 in File S1) and two of these were sequenced and found to have incorporated the sequences at the junctions of the homing unit and the target site (Figure S3 in File S1). The occurrence of partial HR raised concerns that we may have previously incorrectly interpreted cases where no PCR product was obtained with the eGFP-L2/eGFP-R2 primer pair as large deletions arising from NHEJ instead of being the result of extensive but incomplete HR. A further 28 samples that did not yield PCR products were analysed by amplifying across the junctions of the integrated homing unit (Figure S2 and Table S1 in File S1). In one sample, sequences from both junctions were detected while sequences from the right junction only were detected in a further two samples. This indicated that while incomplete HR occurs more frequently that we had considered hitherto, the majority of cases (∼90%) where a PCR product is not obtained by PCR across the cleavage site are appropriately attributed to larger deletions. The incomplete HR products are consistent with a previously-proposed model that HR in *Drosophila* progresses by multiple cycles of strand invasion, synthesis and dissociation of the nascent strand [21].

Unexpectedly, our results show that the propensity for HR repair of double-strand breaks (DSBs) varies depending on the target/nuclease combination. Both the I-SceI and engineered I-OnuI variant nucleases generate DSBs with four-base 3′-overhangs, yet the former is more readily resolved by HR than the latter. The difference could either be a consequence of the immediate sequence context of the DSB or by differences in the rate of nuclease dissociation from the cleaved DNA sequence, thereby delaying access by the repair machinery to the lesion. Studies focused on the factors that control the outcome of targeted DSBs (both repair and homing) are a clear area for future investigation.

### Perspectives into Deploying a HEG Drive System

The results from this first detailed investigation of custom HEGs designs for use in insect HEG gene drive have yielded some insights. On a positive note, they indicate that HEGs designs are active for cleavage and homing in an *in vivo* HEG drive system. The *AGAP004734*-targeted HEGs cleave their intended targets and mediate homing. In addition, these variant endonucleases are sufficiently target-specific such that expression can be tolerated in the male germline without permanent sterility, even when combined with the activity-enhancing, specificity-compromising E178D substitution. However, while stocks bearing the latter can be maintained in laboratory conditions, their fitness may be too impaired to be maintained in a wild population after release.

Our results also identify hitherto unexpected challenges to be addressed, and show that DNA strand hydrolysis catalysed by HEGs is not the sole factor that determines efficient homing. Approaches to HEG redesign currently focus exclusively on optimising cleavage activity and specificity, with the assumption that robust site-specific cleavage should lead to homing at a high frequency. However, very little is known about what selects a DSB repair pathway to fix a break site and it is therefore essential to identify these determinants if we wish to effectively exploit HEGs for control of insect-borne diseases. Our *Drosophila* model provides a uniform, straightforward platform to assess the efficiency of HEG-mediated homing event *in vivo*. Since transgenesis is considerably more difficult in *Anopheles* than *Drosophila*, the model system used here will help to screen for HEG designs with characteristics appropriate to be tested in *Anopheles*.

## Supporting Information

File S1 Combined supplementary information file containing Figures S1–S6 and Table S1. Figure S1, Schematic of an approach to isolating the AGAP004734 gene targeting nucleases using yeast surface display technology. Figure S2, Homing assay. Figure S3, Schematic of PCR primer locations. Figure S4, Sequence alignment of the wild type I-OnuI, two engineered I-OnuI variants cleaving the AGAP004734 gene target (H4734A and H4734B), and their parental enzyme (parental I-OnuI). Figure S5, Positions of substituted residues in the H4734A (a) and H4734B (b) endonucleases. Figure S6, Repair Lesions. Table S1, PCR analysis of repair products of I-OnuI derivatives.
(PDF)
