# Supplementary material for: The Design and In Vivo Evaluation of Engineered I-OnuI-Based Enzymes for HEG Gene Drive
Source: PLoS One. 2013 Sep 10;8(9):e74254. doi: 10.1371/journal.pone.0074254 (PMC3769382; doi:10.1371/journal.pone.0074254)
Supplement: File S1 — Combined supplementary information file containing Figures S1–S6 and Table S1. Figure S1, Schematic of an approach to isolating the AGAP004734 gene targeting nucleases using yeast surface display technology. Figure S2, Homing assay. Figure S3, Schematic of PCR primer locations. Figure S4, Sequence alignment of the wild type I-OnuI, two engineered I-OnuI variants cleaving the AGAP004734 gene target (H4734A and H4734B), and their parental enzyme (parental I-OnuI). Figure S5, Positions of substituted residues in the H4734A (a) and H4734B (b) endonucleases. Figure S6, Repair Lesions. Table S1, PCR analysis of repair products of I-OnuI derivatives. (PDF) [file pone.0074254.s001.pdf]

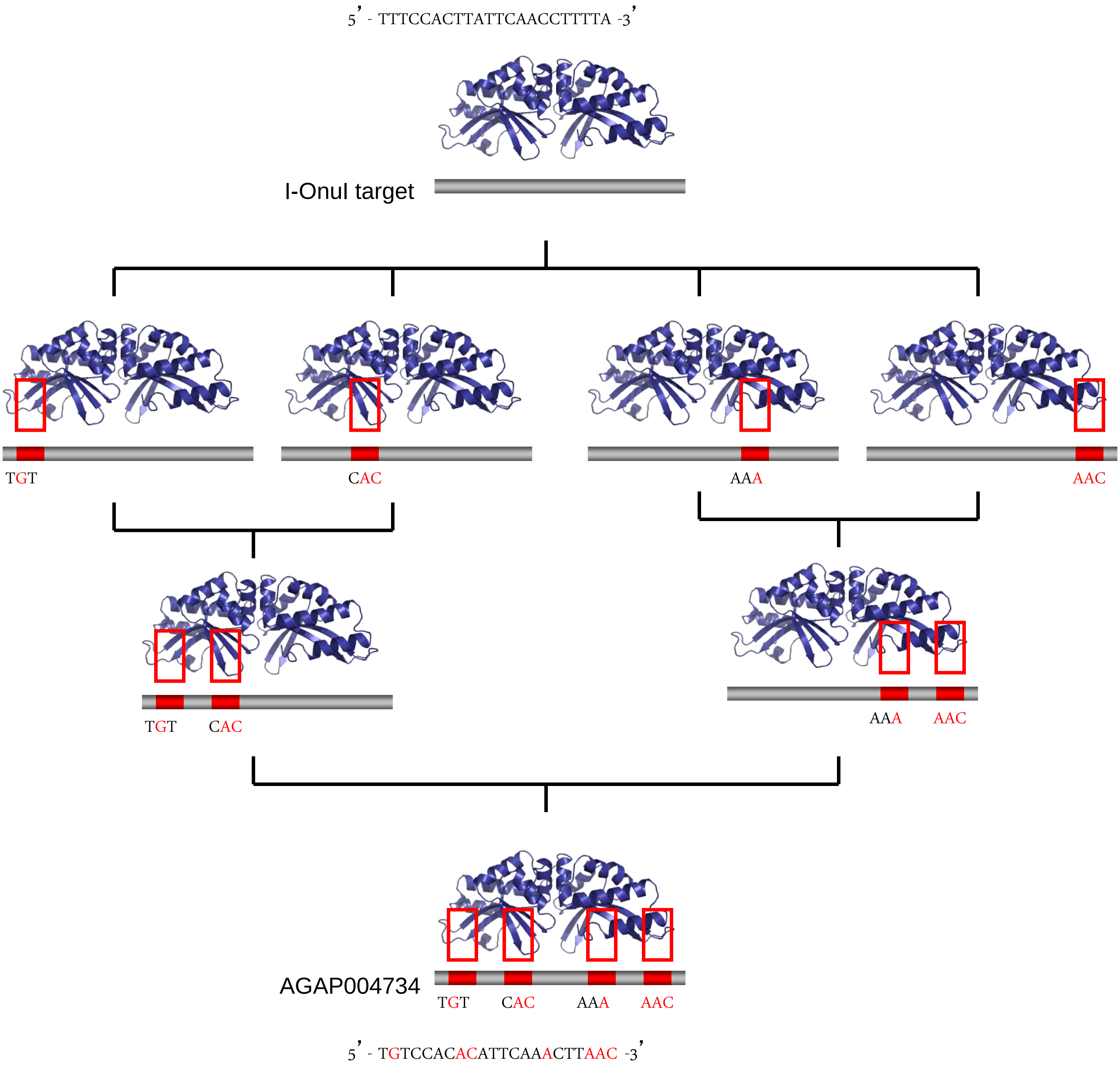

Figure S1. Schematic of an approach to isolating the AGAP004734 gene targeting nucleases using yeast surface display technology. Amino-acid substitutions responsible for cleavage on partially altered target sites were assembled in two steps to create the I-OnuI variants that cleave the full AGAP004734 gene target.

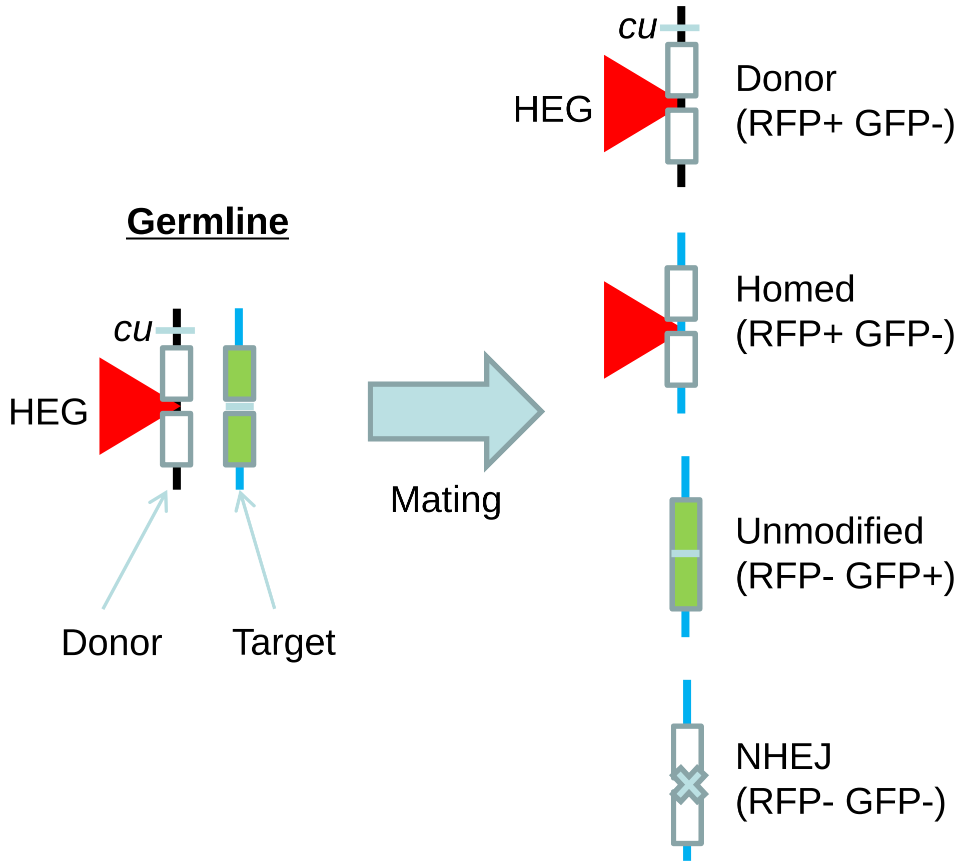

Figure S2. Homing assay In this assay, donor and target constructs were placed at the same φC31 insertion site on homologous chromosomes (the donor and target chromosomes marked black and blue respectively). The target construct contains a GFP open reading frame (ORF) driven by an eye-specific promoter where the GFP ORF is split with an in-frame homing endonuclease recognition site (represented by adjacent green boxes). Transgenics bearing an intact target construct therefore exhibit GFP fluorescence in the eye. The donor construct has a homing endonuclease transcription unit is inserted into the HEG recognition site disrupting the GFP ORF and abolishing GFP fluorescence in the eye (loss of fluorescence represented by the GFP ORF being filled in white). Most constructs also include an RFP marker to allow the HEG insert to be tracked. Expression of the HEG in the germline causes cleavage of its recognition site in the target construct and subsequent repair leads to a number of different outcomes that can be differentiated by fluorescence and phenotypic markers as shown in the figure. The donor and target chromosomes are distinguished either with the linked *cu* marker (applicable with males only because of recombination) or a very closely linked mini-white marker within the donor construct (which is applicable to both sexes). It should be noted that NHEJ repair results in loss of GFP fluorescence in approximately two-thirds of cases only. The remaining third of NHEJ lesions can only be distinguished from unmodified targets by PCR and sequencing.
This figure and legend were taken with permission from (7).

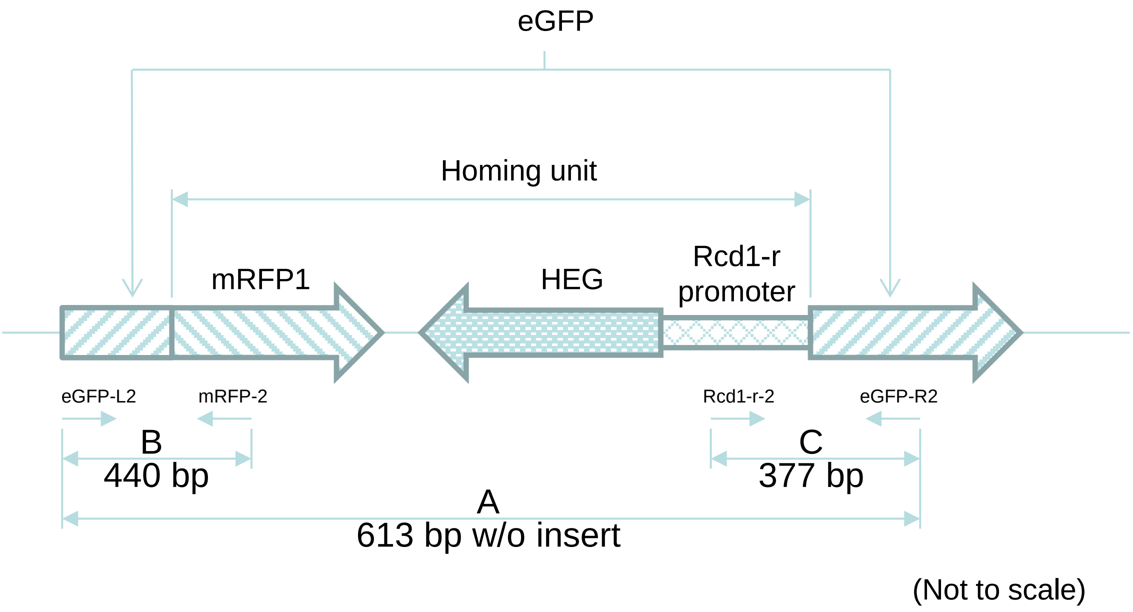

Figure S3 – Schematic of PCR primer locations The figure shows a schematic of the I-OnuI-based homing unit integrated at the AGAP004734 target site (not to scale). The eGFP-L2 and eGFP-R2 primers amplify the region flanking the target site and the sequencing these products are informative for the micro-deletions typical of NHEJ repair although the distance between these primers is too great to yield a product after homing (marked A). Full and/or partial repair of the site via recombination can however be detected with primer pairs eGFP-L2/mRFP-2 and Rcd-1-r2/eGFP-R2 (products marked B and C respectively) that amplify the junctions between the target site flanks and the homing unit.

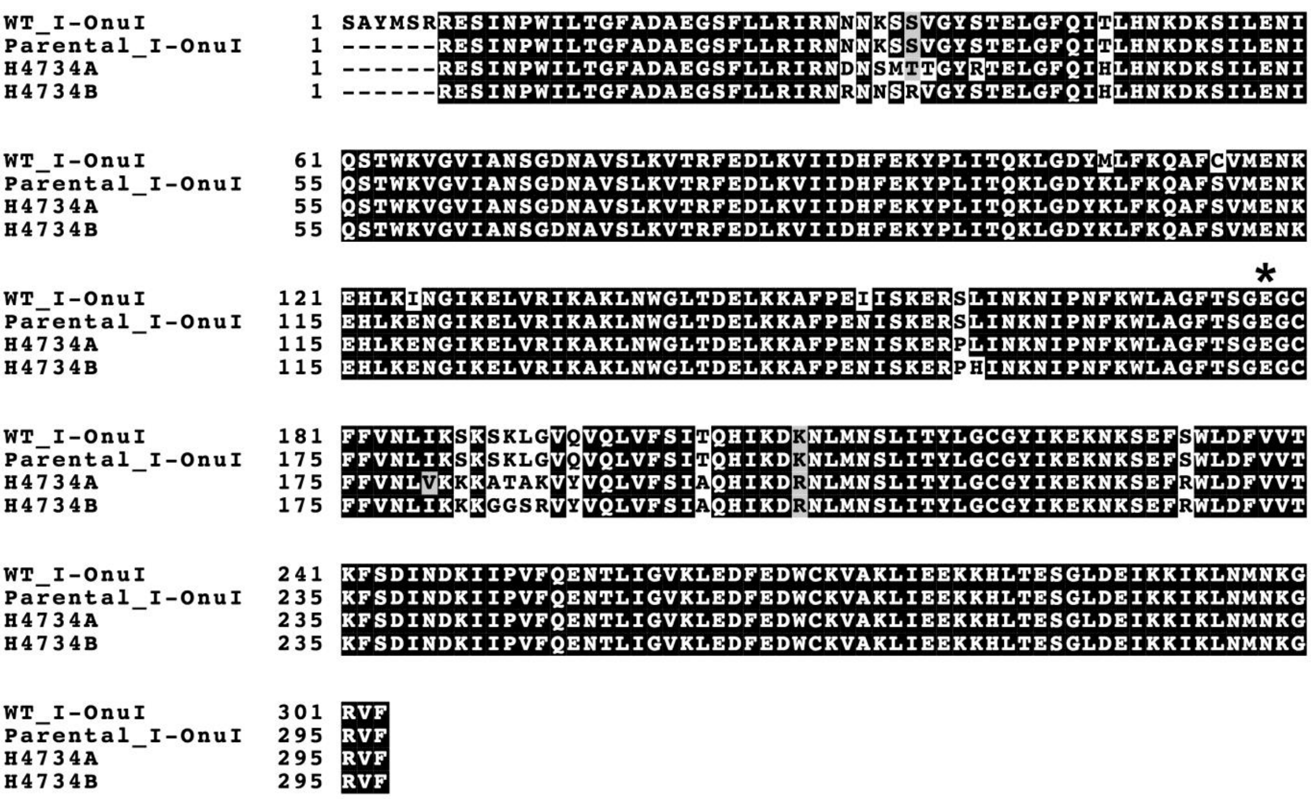

Figure S4. Sequence alignment of the wild type I-OnuI, two engineered I-OnuI variants cleaving the AGAP004734 gene target (H4734A and H4734B), and their parental enzyme (parental I-OnuI). The wild type I-OnuI and the parental I-OnuI differs at 4 residue positions (M108K, C115S, I125E, and I153N). E178 in the active site is marked by an asterisk (*).

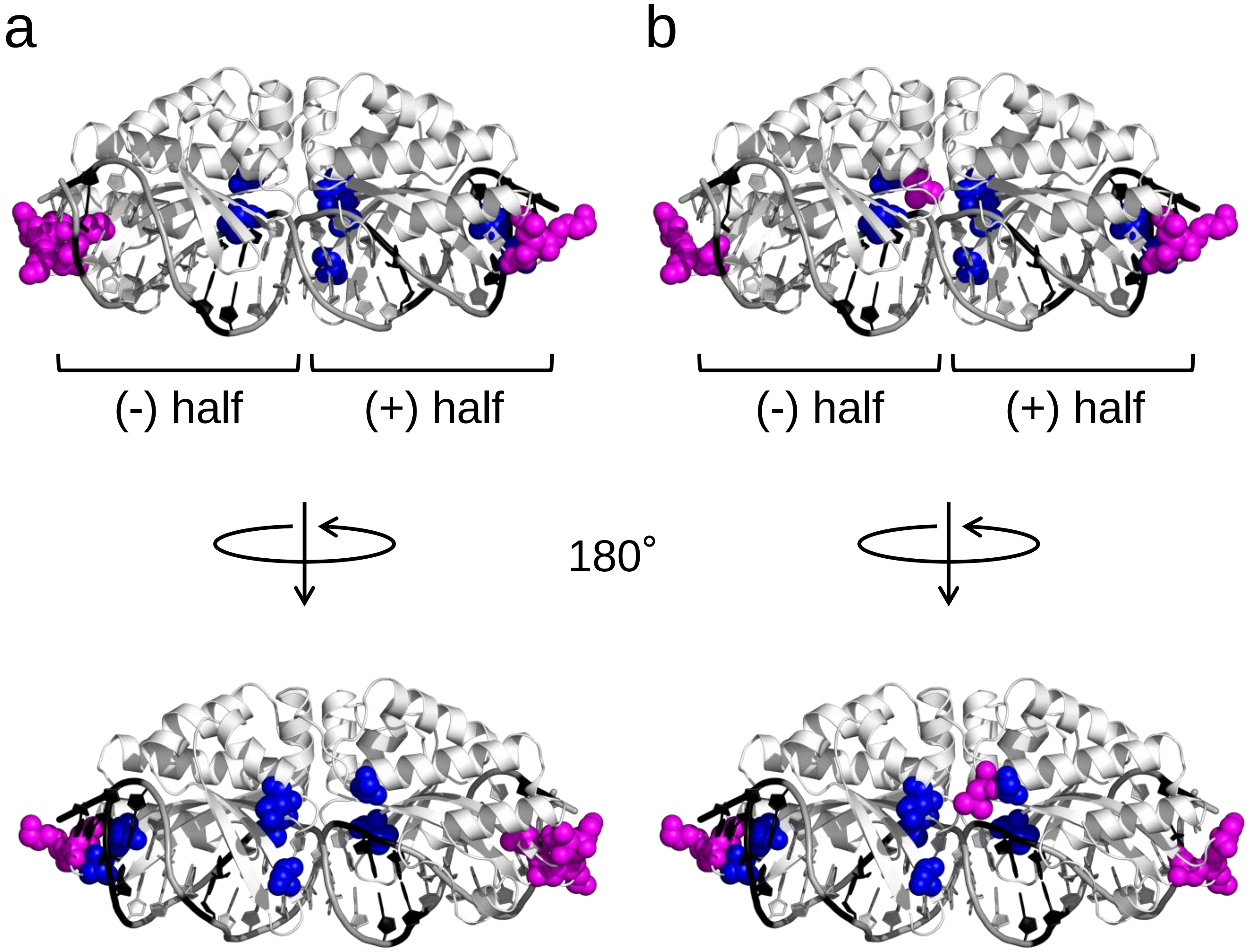

Figure S5. Positions of substituted residues in the H4734A (a) and H4734B (b) endonucleases. Positions where these two variants share the same amino-acid substitution are highlighted in blue. Residues replaced with unique amino acids for individual I-OnuI variants are in magenta. Only the DNA base pairs within the target site for I-OnuI are shown, and base positions where sequences are different between the wild type I-OnuI and the AGAP004734 gene target sites are colored in black.

(A)

H4734A 10   GGTCGCCACCATGCCTAGGGATAAGGGTGTCCAC--**ATTC**AAACTTAACCATAACAGGGTAAT
H4734A 35   GGTCGCCACCATGCCTAGGGATAAGGGTGTCCACA----------------TAACAGGGTAAT
H4734A 9   GGTCGCCACCATGCCTAGGGATAAGGGTGTCCACAC-------------CATAACAGGGTAAT
H4734A 4,8,40   GGTCGCCACCATGCCTAGGGATAAGGGTGTCCACAC----AAACTTAACCATAACAGGGTAAT
H4734A 33   GGTCGCCACCATGCCTAGGGATAAGGGTGTCCACAC**ATTC**-AACTTAACCATAACAGGGTAAT
H4734A 5   GGTCGCCACCATGCCTAGGGATAAGGGTGTCCACAC**ATTC**-----TAACCATAACAGGGTAAT

H4734A* 11   GGTCGCCACCATGCCTAGGGATAAGGGTGTCCACAC**ATTC**--ACTTAACCATAACAGGGTAAT
H4734A* 16   GG----------------------------------**ATTC**AAACTTAACCATAACAGGGTAAT
H4734A* 4,7,15,20   GGTCGCCACCATGCCTAGGGATAAGGGTGTCC----**ATTC**AAACTTAACCATAACAGGGTAAT
H4734A* 24   GGTCGCCACCATGCCTAGGGATAAGGGTGTCCACA-**ATTC**AAACTTAACCATAACAGGGTAAT
H4734A* 27   GGTCGCCACCATGCCTAGGGATAAGGGTGTCCACAC----AAACTTAACCATAACAGGGTAAT
H4734A* 26,29   GGTCGCCACCATGCCTAGGGATAAGGGTGTCCACAC**ATTC**-AACTTAACCATAACAGGGTAAT
H4734A* 28   GGTCGCCACCATGCCTAGGGATAAGGGTGTCCACAC**ATTC**--ACTTAACCATAACAGGGTAAT
H4734A* 17,18   GGTCGCCACCATGCCTAGGGATAAGGGTGTCCACAC**ATTC**----TTAACCATAACAGGGTAAT

H4734B 50   GGTCGCCACCATGCCTAGGGATAAGGGTA-------------------CCATAACAGGGTAAT
H4734B 16,43   GGTCGCCACCATGCCTAGGGATAAGGGTGTCC----**ATTC**AAACTTAACCATAACAGGGTAAT
H4734B 20,41,42   GGTCGCCACCATGCCTAGGGATAAGGGTGTCCAC--**ATTC**AAACTTAACCATAACAGGGTAAT
H4734B 19,48   GGTCGCCACCATGCCTAGGGATAAGGGTGTCCACAC**ATTC**-AACTTAACCATAACAGGGTAAT
H4734B 18   GGTCGCCACCATGCCTAGGGATAAGGGTGTCCACAC**ATTC**----TTAACCATAACAGGGTAAT

H4734B* 22,30,53   GGTCGCCACCATGCCTAGGGATAAGGGTGTC-----**ATTC**AAACTTAACCATAACAGGGTAAT
H4734B* 29,57   GGTCGCCACCATGCCTAGGGATAAGGGTGTCCAC--**ATTC**AAACTTAACCATAACAGGGTAAT
H4734B* 23,25,26,51 GGTCGCCACCATGCCTAGGGATAAGGGTGTCCACA-**ATTC**AAACTTAACCATAACAGGGTAAT
H4734B* 56   GGTCGCCACCATGCCTAGGGATAAGGGTGTCCACA-**ATTC**AAACTTAACCATAACAGGGTAAT
H4734B* 58,60   GGTCGCCACCATGCCTAGGGATAAGGGTGTCCACAC**AT-C**AAACTTAACCATAACAGGGTAAT
H4734B* 55   GGTCGCCACCATGCCTAGGGATAAGGGTGTCCACAC**ATT**-AAACTTAACCATAACAGGGTAAT
H4734B* 54   GGTCGCCACCATGCCTAGGGATAAGGGTGTCCACAC**ATTC**--ACTTAACCATAACAGGGTAAT
H4734B* 21,27   GGTCGCCACCATGCCTAGGGATAAGGGTGTCCACAC**ATTC**----TTAACCATAACAGGGTAAT
H4734B* 59   GGTCGCCACCATGCCTAGGGATAAGGGTGTCCACAC**ATTC**AA-CTTAACCATAACAGGGTAAT

Ref   GGTCGCCACCATGCCTAGGGATAAGGGTGTCCACAC**ATTC**AAACTTAACCATAACAGGGTAAT

(B)

H4734A* 23   GGGATAAGGGTGTCCAC------**TTATTTG**--------AAACTTAACCATAACAGGGTAAT
H4734A* 8,12   GGGATAAGGGTGTCCACAC----ATTC------**TTCAA**AAACTTAACCATAACAGGGTAAT
H4734A* 9   GGGATAAGGGTGTCCACAC----ATTC---**ACATTCAA**AAACTTAACCATAACAGGGTAAT
H4734A* 22   GGGATAAGGGTGTCCACAC----ATTC-**ATACATTCAA**AAACTTAACCATAACAGGGTAAT
H4734B 44   GGGATAAGGGTGTCCACAC**ACAC**ATTC-----------AAACTTAACCATAACAGGGTAAT
H4734B* 28   GGGATAAGGGTGTCCACAC----ATTC-----------AAACTTAACCATAACAGGGTAAT

Figure S6 – Repair Lesions (A) Sequences interpretable as NHEJ events are shown in with the 4-bp cleavage offset sequence displayed in **bold**. The stock and fragment identifiers are indicated on the left of the sequences. (B) Sequences interpretable as insertions at the cleavage site displayed in this panel. The inserted sequences are shown in **bold**. The first entry presumably arose from microhomology-templated repair from another genomic site. The others show duplications of either region flanking the cleavage site.

HEG	H4734A	H4734A*	H4734B	H4734B*
L2-R2 product	30/55 (55%)	122/188 (65%)	12/25 (48%)	18/20 (90%)
No L2-R2 product	23/55 (42%)	66/188 (35%)	11/25 (44%)	2/20 (10%)
Oversized product	2/55 (4%)	-	2/25 (8%)	-
Partial repair	2/17	ND	1/9	0/2

Table S1 – PCR analysis of repair products of I-OnuI derivatives A summary of the analysis of repair lesions by PCR is shown above. L2-R2 product refers to the amplified fragment obtained the eGFP-L2/eGFP-R2 primer pair that spans the cleavage site (fragment A in supplementary figure 4). The row labelled “Partial repair” lists the number of samples and total number of samples analysed in which junctions expected from homologous recombination (fragments B and C in supplementary figure 4) were detected in progeny that did not appear to be homed (GFP-negative, RFP-negative).
